# Retraction Note: Effects of Panax notoginseng saponins on severe acute pancreatitis through the regulation of mTOR/Akt and caspase-3 signaling pathway by upregulating miR-181b expression in rats

**DOI:** 10.1186/s12906-022-03799-4

**Published:** 2022-11-23

**Authors:** Ming-wei Liu, Rui Wei, Mei-xian Su, Hui Li, Tian-wen Fang, Wei Zhang

**Affiliations:** 1 Department of Emergency, the First Hospital Affiliated To Kunming Medical University, 295 Xichang Road, Wu Hua District, Kunming, 650032 China; 2 Intensive Care Unit, The Second Hospital Affiliated To Kunming Medical University, 1 Mayuan Road, Wu Hua District, Kunming, 650106 China; 3 Department of Postgraduate, Kunming Medical University, 1168, Chunrong West Road, Chenggong District, Kunming, 650500 China

**Retraction Note: BMC Complement Altern Med 18, 51 (2018)**

**https://doi.org/10.1186/s12906-018-2118-8**

The Editor has retracted this article because, after publication, concerns were raised with respect to a number of the figures, in particular:

- Panel Beclin1 in Figure 2A appears to contain visual anomalies
- In Figure 5A panel miR-181b overlaps with panel Beclin1mRNA
- In Figure 5A panel mTORmRNA overlaps with panel Beclin1mRNA
- The microscopy images in Figure 7 are also present in Figure 4E of [1]

The Editor therefore no longer has confidence in the veracity of the data presented.

In addition, the rats in this study were euthanized by cervical dislocation which is not an appropriate method for rats in excess of 200 g in mass.

None of the authors have responded to any correspondence from the editor/publisher about this retraction.
